# Statistical methodology for age-adjustment of the GH-2000 score detecting growth hormone misuse

**DOI:** 10.1186/s12874-016-0246-8

**Published:** 2016-10-28

**Authors:** Dankmar Böhning, Walailuck Böhning, Nishan Guha, David A. Cowan, Peter H. Sönksen, Richard I. G. Holt

**Affiliations:** 1 Southampton Statistical Sciences Research Institute, University of Southampton, Southampton, SO17 1BJ UK; 2 Human Development and Health Academic Unit, University of Southampton Faculty of Medicine, IDS Building (MP887), Southampton General Hospital, Tremona Road, Southampton, SO16 6YD UK; 3 Nuffield Division of Clinical Laboratory Sciences, UK Department of Clinical Biochemistry Level 4, University of Oxford, John Radcliffe Hospital Headley Way, Headington, Oxford, OX3 9DU UK; 4 Department of Pharmacy and Forensic Science, Drug Control Centre, King’s College London, 150 Stamford Street, London, SE1 9NH UK

**Keywords:** GH-2000 score, Adjusting for age effects, Meta-analysis of scores, Centring and norming of scores

## Abstract

**Background:**

The GH-2000 score has been developed as a powerful and unique technique for the detection of growth hormone misuse by sportsmen and women. The score depends upon the measurement of two growth hormone (GH) sensitive markers, insulin-like growth factor-I (IGF-I) and the amino-terminal pro-peptide of type III collagen (P-III-NP). With the collection and establishment of an increasingly large database it has become apparent that the score shows a positive age effect in the male athlete population, which could potentially place older male athletes at a disadvantage.

**Methods:**

We have used results from residual analysis of the general linear model to show that the residual of the GH-2000 score when regressed on the mean-age centred age is an appropriate way to proceed to correct this bias. As six GH-2000 scores are possible depending on the assays used for determining IGF-I and P-III-NP, methodology had to be explored for including six different age effects into a unique residual. Meta-analytic techniques have been utilized to find a summary age effect.

**Results:**

The age-adjusted GH-2000 score, a form of residual, has similar mean and variance as the original GH-2000 score and, hence, the developed decision limits show negligible change when compared to the decision limits based on the original score. We also show that any further scale-transformation will not change the adjusted score. Hence the suggested adjustment is optimal for the given data. The summary age effect is homogeneous across the six scores, and so the generic adjustment of the GH-2000 score formula is justified.

**Conclusions:**

A final revised GH-2000 score formula is provided which is independent of the age of the athlete under consideration.

## Background

Growth hormone is a powerful anabolic agent of considerable therapeutic value but also misused in sport for its anabolic and lipolytic properties [1]. In order to preserve the fairness of competition, its use is prohibited by the World Anti-Doping Agency [2] and there is a need for methods to detect its misuse. Two methods are presently available and approved by the World Anti-Doping Agency (WADA); the isoform test developed by Bidlingmaier et al. [3]) (see also [4]) and the GH-2000 biomarker test developed by the GH-2000 and GH-2004 projects [5]. The latter method depends upon the measurement of two growth hormone (GH) sensitive markers, insulin-like growth factor-I (IGF-I) and the amino-terminal pro-peptide of type III collagen (P-III-NP), both of which rise in response to exogenous GH administration [6, 7]. The measured concentrations of the biomarkers are combined in sex-specific and age-adjusted discriminant functions, which allow for the calculation of a score (the GH-2000 score) on which basis the compliance of the sample’s analytical result is determined. The age correction is required because GH secretion and markers of its action rise during childhood and reach a peak in early adulthood before declining at a rate of ~14 % per decade [8]. Without an adjustment for age, younger athletes are placed at a disadvantage. For IGF-I and P-III-NP, a model in which the log of the marker level decreased linearly with the reciprocal of age fitted the data from 693 elite athlete marker levels well, over the range of ages studied [9] and a term with the reciprocal for age was included in the GH-2000 score [10]. The inverse term for age is designed to adjust for age so that the score becomes independent of age. This is important in order to make the test applicable to athletes of all ages.

The initial development of the GH-2000 score was based on immunoassays that are no longer commercially available. Although the original discriminant function has remained unchanged, the decision limits have been updated as further experience was accumulated and new assays became available [5, 11]. Currently, there are three IGF-I assays and two P-III-NP assays approved by WADA.

The IGF-I assays used in this study were:

- Liquid chromatography-tandem mass spectrometry (LC-MS/MS)
- Immunotech A15729 IGF-I IRMA (Immunotech SAS, Marseille, France)
- and Immunodiagnostic Systems iSYS IGF-I (Immunodiagnostics Systems Limited, Boldon, UK)

The P-III-NP assays used in this analysis were:

- UniQ™ P-III-NP RIA (Orion Diagnostica, Espoo, Finland)
- Siemens ADVIA Centaur P-III-NP (Siemens Healthcare Laboratory Diagnostics, Camberley, UK).

For more details and background on these assays see Holt et al. [5].

As these assays do not give identical results, different GH-2000 scores are obtained with each of the combinations and this means that the decision limits are different, depending on the assay pair used.

Recent analysis of a combined database of 998 male and 931 female elite athletes [5] provides evidence that the score is independent of age for the female population whereas it shows a linear dependence for male athletes. This indicates that the original inverse term for age over-corrects for the natural decline in GH markers thereby potentially placing older athletes at a disadvantage.

The combined database contains blood samples of athletes collected at various sporting events including the 2011 International Association of Athletics Federations (IAAF) World Athletics Championships in Daegu, South Korea, in the following abbreviated as the Daegu sample.

**Fig. 1:**
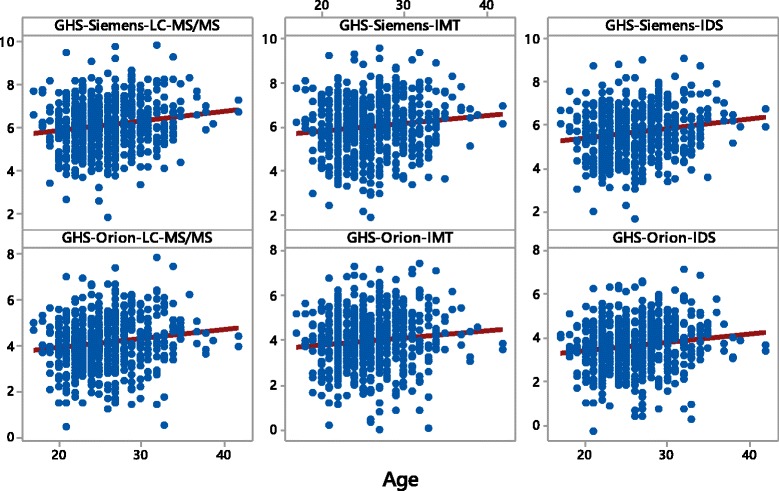
Scatterplots with regression lines for the six GH-2000 scores (GHS) available of all *male* athletes in the Daegu sample: Siemens-LC-MS/MS (*top-left*), Siemens-Immunotech (*top-middle*), Siemens-IDS (*top-right*), Orion-LC-MS/MS (*bottom-left*), Orion-Immunotech (*bottom-middle*), Orion-IDS (*bottom-right*)

Figure 1 shows the scores and their relationship to age in 597 male athletes competing in Daegu. There are 6 scores as there are 3 assays for IGF-I (LC-MS/MS, Immunotech, IDS) and 2 for P-IIIN-P (Siemens-Centaur, Orion). It is clear from Fig. 1 that in all GH-2000 scores there is a positive age dependency as all linear regression lines show a significant age-effect. This positive age dependency is also seen in nonparametric regression of the GH-2000 score on age and, hence, is of structural nature and not caused by artefacts such as outlying observations. There is no age effect on the GH-2000 scores for the female population of the Daegu sample indicating that the original age correction term performs well in a new independent database (data not shown).

The purpose of this paper is to suggest and discuss statistical methodology for adjusting the existing male GH-2000 score for the undesirable age-effect.

## Methods

### The GH-2000 score

The GH-2000 score has been developed in Powrie et al. [10], Erotokritou-Mulligan et al. [11] and Holt et al. [5]. It has the theoretical or model form

1 $$ \mathrm{G}\mathrm{H}2000\ \mathrm{score}={\beta}_0+{\beta}_1\  \log\ \left(\mathrm{I}\mathrm{G}\mathrm{F}\hbox{-} \mathrm{I}\right) + {\beta}_2\  \log\ \left(\mathrm{P}\hbox{-} \mathrm{I}\mathrm{I}\mathrm{I}\hbox{-} \mathrm{N}\mathrm{P}\right) + {\beta}_3/\mathrm{age} $$

where the coefficients *β*_0_, *β*_1_, *β*_2_, *β*_3_ have different values for male and female athletes. When coefficients are replaced by estimates the GH-2000 score for male athletes is

2 $$ \mathrm{G}\mathrm{H}2000\ \mathrm{score}=-6.586+2.100\  \log\ \left(\mathrm{I}\mathrm{G}\mathrm{F}\hbox{-} \mathrm{I}\right) + 2.905\  \log\ \left(\mathrm{P}\hbox{-} \mathrm{I}\mathrm{I}\mathrm{I}\hbox{-} \mathrm{N}\mathrm{P}\right) - 101.737/\mathrm{age} $$

and for female athletes

3 $$ \mathrm{G}\mathrm{H}2000\ \mathrm{score}=-8.459+2.195\  \log\ \left(\mathrm{I}\mathrm{G}\mathrm{F}\hbox{-} \mathrm{I}\right) + 2.454\  \log\ \left(\mathrm{P}\hbox{-} \mathrm{I}\mathrm{I}\mathrm{I}\hbox{-} \mathrm{N}\mathrm{P}\right) - 73.666/\mathrm{age} $$

As we have seen in the previous section, the GH-2000 score shows positive age-dependency for the male population. Adjusting for the age-effect will be considered in the next section.

### The basics of adjustment

Consider a response *Y* (in our case the GH-2000 score) and an effect *x* (in our case the age of an athlete). Suppose that the response *Y* is related to *x* by a linear regression model

4 $$ E(Y) = \alpha + \beta x $$

Then, the least-squares estimate of *β* in (4) is given by

5 $$ \widehat{\beta}=\frac{{\displaystyle \sum_{i=1}^n\Big({Y}_i}-\overline{Y}\Big)\left({x}_i-\overline{x}\right)}{{\displaystyle \sum_{i=1}^n\Big({x}_i-\overline{x}}\Big){}^2} $$

where the pairs (*Y*_*i*_, *x*_*i*_) represent the *n* sample values of *Y* and *x*. On this basis we are able to construct a response ${Y}^{*} = Y - \widehat{\beta}x$ adjusting for *x*.

The adjusted response *Y* ^*^is independent of *x* as the following analysis shows. This can be found in most books on regression but it is mentioned here for completeness. Consider the least-squares-estimate of *β* ^*^ in (6)

6 $$ E\left({Y}^{*}\right) = {\alpha}^{*} + {\beta}^{*}x. $$

This least-squares estimate of *β* ^*^ is provided as zero as equation (7) shows:

7 $$ \begin{array}{c}{\widehat{\beta}}^{*}=\frac{{\displaystyle \sum_{i=1}^n\left({Y}_i^{*}-\overline{Y*}\right)\left({x}_i-\overline{x}\right)}}{{\displaystyle \sum_{i=1}^n{\left({x}_i-\overline{x}\right)}^2}}=\frac{{\displaystyle \sum_{i=1}^n\Big({Y}_i-\widehat{\beta}{x}_i}-\left(\overline{Y},-,{\widehat{\beta}}^{*}\right)\Big)\left({x}_i-\overline{x}\right)}{{\displaystyle \sum_{i=1}^n{\left({x}_i-\overline{x}\right)}^2}}\\ {}=\frac{{\displaystyle \sum_{i=1}^n\Big({Y}_i-\overline{Y}}\left)\left({x}_i-\overline{x}\right)-\widehat{\beta}{\displaystyle \sum_{i=1}^n\Big({x}_i-\overline{x}}\right){}^2}{{\displaystyle \sum_{i=1}^n{\left({x}_i-\overline{x}\right)}^2}}=\frac{{\displaystyle \sum_{i=1}^n\Big({Y}_i-}\overline{Y}\Big)\left({x}_i-\overline{x}\right)}{{\displaystyle \sum_{i=1}^n{\left({x}_i-\overline{x}\right)}^2}}-\widehat{\beta}\frac{{\displaystyle \sum_{i=1}^n\Big(}{x}_i-\overline{x}\Big){}^2}{{\displaystyle \sum_{i=1}^n{\left({x}_i-\overline{x}\right)}^2}}=0.\end{array} $$

Hence *Y* ^*^ is independent of *x*. A more general result is provided in Appendix 1.

Next, we suggest considering an adjustment of the form

8 $$ {Y}^{*} = Y - \widehat{\beta}\left(x-\overline{x}\right). $$

The benefit of this adjustment (8) lies in the fact that the adjusted score *Y* ^*^ remains on the *same level* as the original score *Y* as

9 $$ {\overline{Y}}^{*}=\overline{Y}-\widehat{\beta}\left(\overline{x}-\overline{x}\right)=\overline{Y}. $$

The process of considering $x-\overline{x}$ is called *centering*. Sometimes also *norming* is considered in addition to centering which is $\left(x-\overline{x}\right)/sd(x)$ where $sd(x)=\sqrt{\frac{1}{n-1}{\displaystyle \sum_{i=1}^n{\left({x}_i-\overline{x}\right)}^2}}.$ We are not considering norming here as this will not lead to any further adjustment. To see this, we consider any scale transformation *ax* of *x*. The original model *E* (*Y*) = *α* + *βx* becomes now *E* (*Y*) = *α* ^*^ + *β* ^*^ *x* ^*^, where *x* ^*^ = *ax*. Then, least squares estimates can be found as

10 $$ {\widehat{\beta}}^{*}=\frac{{\displaystyle \sum_{i=1}^n\Big({Y}_i-}\overline{Y}\Big)\left({x}_i^{*}-\overline{x^{*}}\right)}{{\displaystyle \sum_{i=1}^n{\left({x}_{{}_i}^{*}-\overline{x^{*}}\right)}^2}}=\frac{{\displaystyle \sum_{i=1}^n\Big({Y}_i-}\overline{Y}\Big)\left({x}_i-\overline{x}\right)a}{{\displaystyle \sum_{i=1}^n{\left({x}_i-\overline{x}\right)}^2{a}^2}}=\frac{1}{a}\frac{{\displaystyle \sum_{i=1}^n\Big({Y}_i-}\overline{Y}\Big)\left({x}_i-\overline{x}\right)}{{\displaystyle \sum_{i=1}^n{\left({x}_i-\overline{x}\right)}^2}}=\frac{1}{a}\widehat{\beta} $$

Hence the adjusted response (11)

11 $$ {Y}^{*} = Y - {\widehat{\beta}}^{*}{x}^{*} = Y - \left(\frac{1}{a}\widehat{\beta}\right) ax = Y - \widehat{\beta}x $$

is indeed identical to the original adjustment $Y - \widehat{\beta}x$ and does not lead to anything new. A more general result is provided in Appendix 2. Hence we stay with the adjustment ${Y}^{*} = Y - \widehat{\beta}\left(x-\overline{x}\right)$’, provided in (8), as the final form of adjustment.

### Adjusting the GH-2000 score

To adjust the GH-2000 score, we consider the regression of the GH-2000 score on age. Table 1 shows 6 age-effects for the 6 GH-2000 scores (as there are 2 assays for measuring P-III-NP and 3 assays for measuring IGF-I).

**Table 1:** Estimated *β*-coefficients of the age-effects for the six GH-2000 scores and their associated standard errors

GH-2000 score		$\widehat{\beta}$ age	S.E.
P-III-NP assay	IGF-I assay		
Siemens	LC-MS/MS	0.0418	0.0082
Siemens	Immunotech	0.0261	0.0086
Siemens	IDS	0.0359	0.0070
Orion	LC-MS/MS	0.0363	0.0077
Orion	Immunotech	0.0202	0.0085
Orion	IDS	0.0318	0.0077

For simplicity and ease of use by the anti-doping laboratories, it is important that we do not create an age adjustment for each assay pairing. Thus we need to include the age adjustment within the generic GH-2000 score (independent of the specific assay pairing used). To accomplish this task we have applied ideas from meta-analysis. We consider each GH-2000 score using a specific assay combination as a realisation from multiple possible assay combinations.

This is similar to a meta-analysis approach in which studies aiming to estimate a certain effect are considered as realisation from a universe of possible studies.

Hence we use

12 $$ \overline{\beta}={\displaystyle \sum_{i=1}^k{w}_i{\widehat{\beta}}_i}/{\displaystyle \sum_{i=1}^k{w}_i} $$

where *k* = 6 is the number of different assay combinations used and ${\widehat{\beta}}_i$ is the estimated age effect, and *w*_*i*_ is the inverse of the estimated variance (the squared values in column 3 of Table 1). Hence $\overline{\beta}$ is an average of the estimated effect.

## Results

**Fig. 2:**
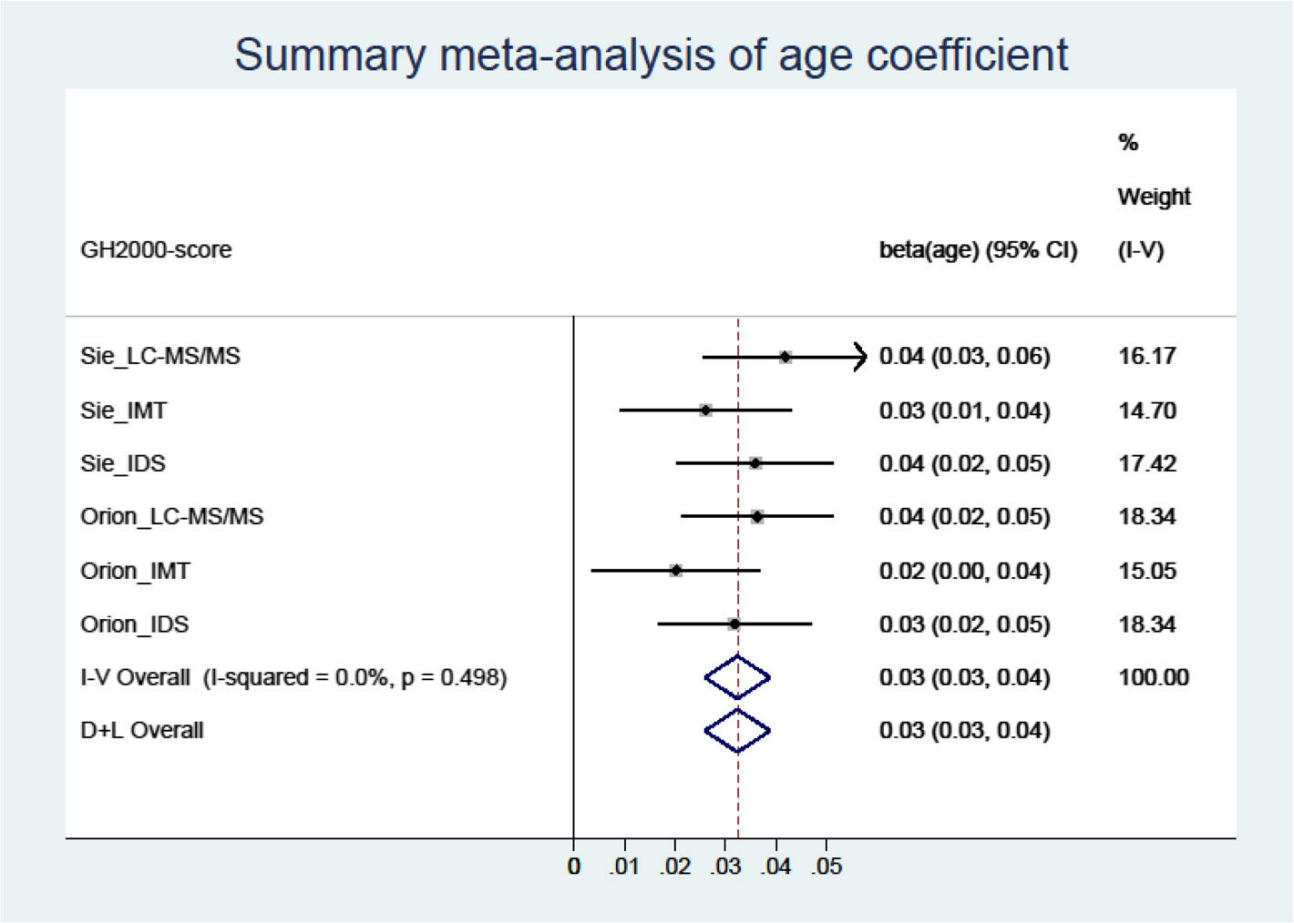
Meta-analytic results for the six age-effects of the GH-2000 scores on age (I-V stands for overall inversely weighted and provides the summary estimate of the age-affect); more details are given in the appendix, the arrow-to-right indicates that the right confidence limit falls outside the plotting area

In our case, we find $\overline{\beta}$ = 0.032. Figure 2 shows this analysis graphically. As all assay-specific age effects are similar in their standard error, all weights are similar. More details on the meta-analysis approach are given in Appendix 3.

To investigate the appropriateness of the meta-analytic weighted average approach (are the age-effects for the six scores similar enough to be validly combined in a weighted average?) a heterogeneity analysis was performed. The *X* ^2^-test of homogeneity ${\chi}^2={\displaystyle \sum_{i=1}^6\frac{{\left({\widehat{\beta}}_i-\overline{\beta}\right)}^2}{\operatorname{var}\left({\widehat{\beta}}_i\right)}}$ delivers a value of 4.37 which has a non-significant *p*-value of 0.498 by 5 df. Hence the approach we have taken is justified (details are given in the Appendix 3).

From the meta-analysis, we achieve the formula for the male athletes:

$$ \mathrm{G}\mathrm{H}\hbox{-} 2000\ \mathrm{score}\hbox{-} \mathrm{a}\mathrm{d}\mathrm{j} = \mathrm{G}\mathrm{H}\hbox{-} 2000\ \mathrm{score}\ \hbox{--}\ 0.032\ \left(\mathrm{age}\ \hbox{-}\ 25.09\right) $$

As the mean age for male athletes is 25.09 years and the GH-2000 is calculated as:

$$ \mathrm{G}\mathrm{H}\hbox{-} 2000\ \mathrm{score} = - 6.586 + 2.905\  \log \left(\mathrm{P}\hbox{-} \mathrm{I}\mathrm{I}\mathrm{I}\hbox{-} \mathrm{N}\mathrm{P}\right) + 2.100\  \log \left(\mathrm{I}\mathrm{G}\mathrm{F}\hbox{-} \mathrm{I}\right)\ \hbox{--}\ 101.737/\mathrm{age} $$

the *adjusted score* formula becomes:

13 $$ \mathrm{G}\mathrm{H}-2000\ \mathrm{score}\hbox{-} \mathrm{a}\mathrm{d}\mathrm{j} = - 5.783 + 2.905\  \log \left(\mathrm{P}\hbox{-} \mathrm{I}\mathrm{I}\mathrm{I}\hbox{-} \mathrm{N}\mathrm{P}\right) + 2.100\  \log \left(\mathrm{I}\mathrm{G}\mathrm{F}\hbox{-} \mathrm{I}\right)\ \hbox{--}\ 101.737/\mathrm{age}\ \hbox{--}\ 0.032\ \mathrm{age}. $$

**Fig. 3:**
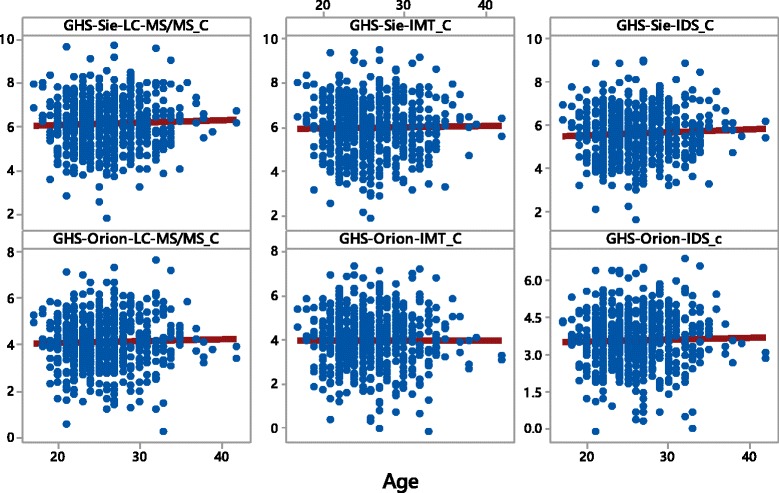
Scatterplots with regression lines for the six *age-adjusted* GH-2000 scores (GHS) of all *male* athletes in the Daegu-sample in the order of their appearance: Siemens-LC-MS/MS (*top-left*), Siemens-Immunotech (*top-middle*), Siemens-IDS (*top-right*), Orion-LC-MS/MS (*bottom-left*), Orion-Immunotech (*bottom-middle*), Orion-IDS (*bottom-right*)

Figure 3 shows a scatterplot of the six *age-adjusted* GH-2000-scores. It clearly shows that the age-effect is removed as it is expected from the above theory.

### Effect on the current WADA decision limits

Although this adjustment will lead to changes in the individual GH-2000 score of an athlete, it has negligible effect on the decision limits. The decision limits are most important in practice as they provide the cut-off value above which the athlete’s GH-2000 score value is considered to be positive. Following Holt et al. [5] these are constructed using the 1 in 10,000 false positive rate as

14 $$ \mathrm{D}\mathrm{L}=\overline{y}+3.72s+u $$

where $\overline{y}$ and *s* are mean and standard deviation of the respective GH-2000 score. *u* is a sample uncertainly term defined as

15 $$ u=\sqrt{\frac{s^2}{n}\left(1+\frac{3.72^2}{n}\right)} $$

where *n* is the sample size. Table 2 shows the details, in particular, a comparison between GH-2000 scores with and without adjustment

**Table 2:** Descriptive statistics including decision limits for the 6 unadjusted and adjusted GH-2000 scores

Assay pair			*n*	mean	s^a^	Mean + 3.72*s	u^b^	DL^c^
P-III-NP	IGF-I							
Siemens	LC-MS	Unadjusted Adjusted	947 947	6.5393 6.5382	1.2412 1.2424	11.1566 11.1599	0.1872 0.1874	11.34 11.35
Siemens	Immunotech	Unadjusted Adjusted	971 971	6.4292 6.4287	1.3189 1.3284	11.3355 11.3703	0.1965 0.1979	11.53 11.57
Siemens	IDS	Unadjusted Adjusted	970 970	5.9935 5.9931	1.1925 1.1954	10.4296 10.4400	0.1777 0.1782	10.61 10.62
Orion	LC-MS	Unadjusted Adjusted	966 966	4.7062 4.7047	1.2902 1.2976	9.5057 9.5318	0.1927 0.1938	9.70 9.73
Orion	Immunotech	Unadjusted Adjusted	999 999	4.5984 4.5984	1.3925 1.4077	9.7785 9.8350	0.2045 0.2068	9.98 10.04
Orion	IDS	Unadjusted Adjusted	992 992	4.1614 4.1611	1.2522 1.2610	8.8196 8.8520	0.1846 0.1859	9.00 9.04

^a^s is the standard deviation, ^b^u is the uncertainty correction (13), ^c^DL is the decision limit (12)

### Distribution of adjusted GH-2000 scores

**Fig. 4:**
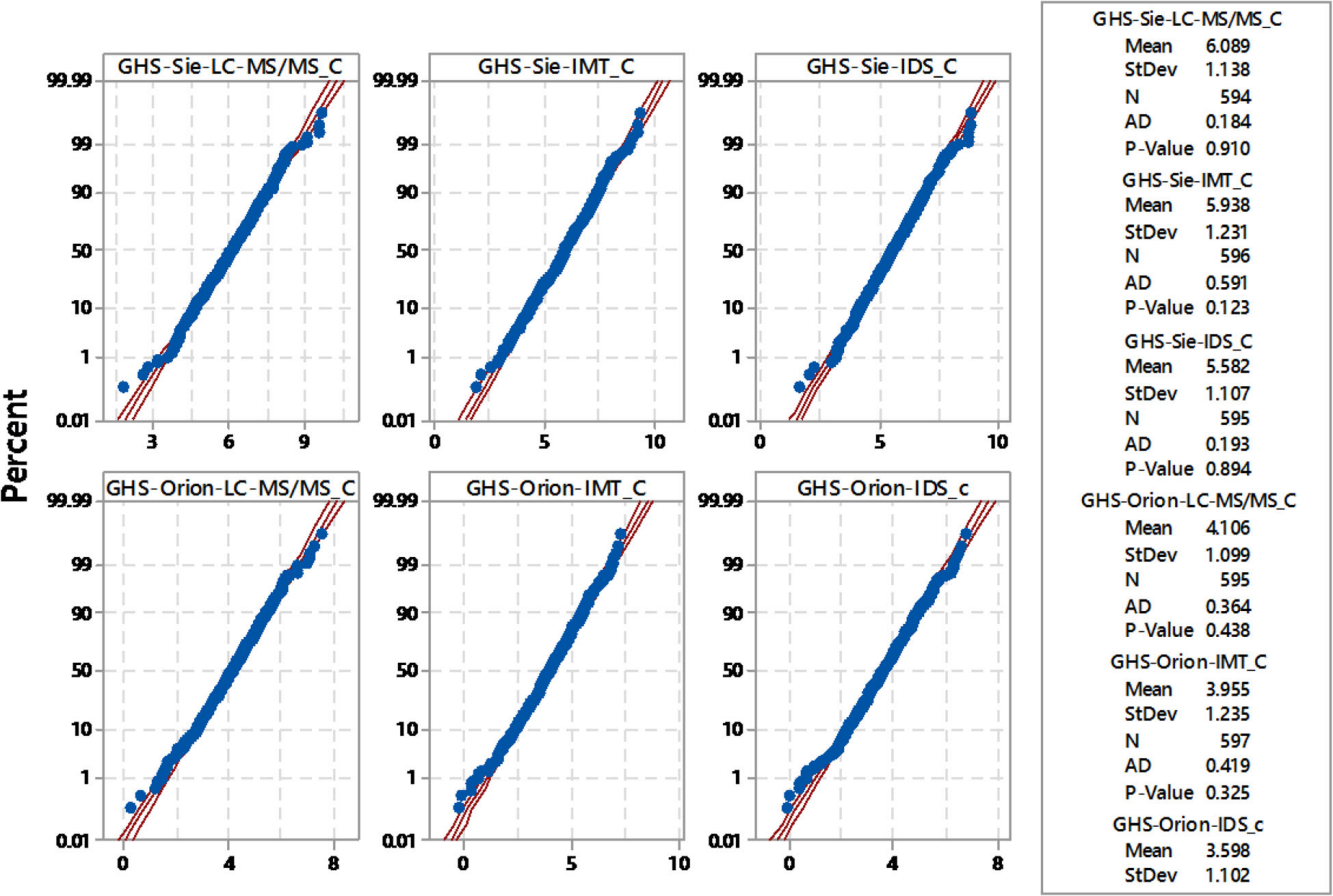
Probability plots for the six GH-2000 scores (GHS) adjusted for age; AD stand for Anderson-Darling test of normality and the *P*-value refers to the null-hypothesis of normality so that values larger than 0.05 do not lead to rejection of normality

The construction of the decision limits for GH-2000 biomarker methodology is dependent on a normal distribution of GH-2000 scores among clean athlete. This was assessed using probability plotting and the Anderson-Darling test for normality which provided clear evidence that all six scores were normally distributed (Fig. 4).

## Discussion

We are suggesting this adjustment for the male elite athlete population only, as the female population does not show age dependency. It could be demonstrated that the proposed adjustment of the GH-2000 score removes the positive age dependency.

Furthermore, the age-adjustment of the score is also beneficial with respect to the normality of the scores as the probability plot in Fig. 4 shows that all scores appear to be normal.

The GH-2000 and GH-2004 teams have previously published the rationale and background to the development of decision limits for the GH-2000 biomarker detection method [5, 10].

It was always envisaged that a dynamic approach would be taken towards refining the decision limits as further data became available. Our recent investigations have shown that the age-adjustment in the male discriminant function, which was derived the original GH-2000 cross-sectional elite athlete study [9, 10], over-corrects for age in male athletes in our more recent cohorts. The effect of this over-correction is to place older male athletes at a slight disadvantage compared with their younger peers, for whom the sensitivity of the test is reduced. The original age correction for women remained valid in the later cohorts. We have used the most recent dataset, on which the current decision limits are based, to add a smaller further adjustment to the discriminant function to address this issue.

When undertaking this analysis, we used several principles to guide out work: 1) we wanted to ensure that the updated male discriminant function was unaffected by age in order to make the test equally fair and effective for athletes of all ages; 2) the change in age correction would have a minimal effect on the current decision limits; and 3) a single age adjustment could be applied for all assay pairings. In order to minimise the effect on the current decision limits, we used a method that centred the data. By doing so the mean GH-2000 scores were virtually unaffected. There was a trivial change to the SDs and consequently the decision limits, which are based on the mean and SD, were unchanged. The age adjustment varies slightly by assay pairing and in order to overcome this, we adapted meta-analytical methodology to derive a common age adjustment for all the combinations. There was no evidence of heterogeneity between the assay pairings and each contributed to the final adjustment equally, providing support for this approach.

## Conclusion

In conclusion, we have created a small further age adjustment for male athletes to correct the age bias introduced with the original discriminant formula. This has no effect on the decision limits and should be easily introduced into anti-doping testing.

## Figures and Tables

**Fig. 5:**
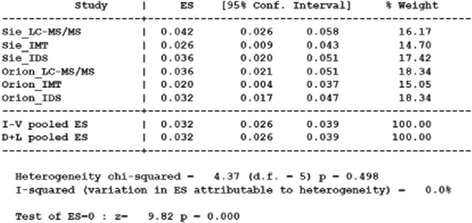
Meta-analytic results produced by the add-on package METAN of STATA14 for the six age-effects of the GH2000 scores on age (I-V stands for overall inversely weighted and provides the summary estimate of the age-affect)

## Appendix 1

### Independence of residuals from model covariates

Consider a general linear model *Y* = X *β* + *ε* where *Y* is a *n*-vector of responses, *X* is the design matrix containing the *n*-values of *p* covariates, *ε* is a *n*-vector of errors, and *β* is a *p-*vector of unknown parameters. Then, the least-squares estimate for *β* is given as

A1 $$ \widehat{\beta}={\left({X}^TX\right)}^{-1}{X}^TY $$

and the vector of residuals ${Y}^{*}=Y-X\widehat{\beta}$. Regressing *Y* ^*^ on *X* leads to the general linear model

A2 $$ {Y}^{*}=X{\beta}^{*}+{\varepsilon}^{*} $$

and the least-squares estimate of *β* ^***^ is given as

A3 $$ \begin{array}{c}{\widehat{\beta}}^{*}={\left({X}^TX\right)}^{-1}{X}^T{Y}^{*}={\left({X}^TX\right)}^{-1}{X}^T\left(Y-X\widehat{\beta}\right)\\ {}={\left({X}^TX\right)}^{-1}{X}^T\left[Y-X{\left({X}^TX\right)}^{-1}{X}^TY\right]\\ {}={\left({X}^TX\right)}^{-1}{X}^TY-{\left({X}^TX\right)}^{-1}{X}^TX{\left({X}^TX\right)}^{-1}{X}^TY\\ {}={\left({X}^TX\right)}^{-1}{X}^TY-{\left({X}^TX\right)}^{-1}{X}^TY=0,\end{array} $$

showing that the residuals are independent from all covariates included in the model. See also Sen and Srivastava [12].

## Appendix 2

### Invariance of the effect estimates with respect to scale transformations

Consider a general linear model *Y* = *X β* + *ε* where *Y* is a *n*-vector of responses, *X* is the design matrix containing the *n*-values of *p* covariates, *ε* is a *n*-vector of errors, and *β* is a *p-*vector of unknown parameters. Now let *A* be an invertible *p* × *p* matrix and *XA* the associated scale-transformation of the design matrix. Then, the least-squares estimate of the transformed model *Y* = *XAβ* ^***^ + *ε* ^***^ is given as

B1 $$ \begin{array}{l}{\widehat{\beta}}^{*}={\left({A}^T{X}^TXA\right)}^{-1}{A}^T{X}^TY={\left({X}^TXA\right)}^{-1}{\left({A}^T\right)}^{-1}{A}^T{X}^TY\\ {}={\left({X}^TXA\right)}^{-1}{X}^TY={A}^{-1}{\left({X}^TX\right)}^{-1}{X}^TY.\end{array} $$

It follows that the residual with respect to the scale-transformed design matrix

B2 $$ \begin{array}{l}Y-XA{\widehat{\beta}}^{*}=Y-XA{A}^{-1}{\left({X}^TX\right)}^{-1}{X}^TY\\ {}=Y-X{\left({X}^TX\right)}^{-1}{X}^TY=Y-X\widehat{\beta}\end{array} $$

is identical to the residual of the untransformed design matrix. See also [12]. As a consequence norming (for example by standard deviations of covariates) of the covariates will not change the residuals.

## Appendix 3

### Heterogeneity analysis

Here we give more details on the meta-analytic approach we have taken. Figure 5 shows the various elements involved in the meta-analysis.

The basic elements are the six GH2000 scores with their age-effects and weights according to the inverse variance (similar variance). The two bottom rows show the summary effect with and without heterogeneity. Both are virtually identical, as there is no heterogeneity (*I* ^2^ = 0, no variation due to heterogeneity). In case there is heterogeneity we would consider the DerSimonian-Laird approach which incorporates heterogeneity into the weighting scheme. In our case, both analyses lead to the same result. All analysis is based on the add-on package METAN of the statistical software STATA14 [13].

## Abbreviations

GH-2000 score: Growth hormone 2000 score
DL: Decision limit
IGF-I: Insulin-like growth factor-I
P-III-NP: Amino-terminal pro-peptide of type III collagen
LC-MS/MS: Immunotech, IDS are assays to measure IGF-I
Siemens-Centaur: Orion are assays to measure P-III-NP
