# Erratum to: Statistical methodology for age-adjustment of the GH-2000 score detecting growth hormone misuse

**DOI:** 10.1186/s12874-016-0262-8

**Published:** 2016-11-28

**Authors:** Dankmar Böhning, Walailuck Böhning, Nishan Guha, David A. Cowan, Peter H. Sönksen, Richard I. G. Holt

**Affiliations:** 1 Southampton Statistical Sciences Research Institute, University of Southampton, Southampton, SO17 1BJ UK; 2 Human Development and Health Academic Unit, University of Southampton Faculty of Medicine, IDS Building (MP887), Southampton General Hospital, Tremona Road, Southampton, SO16 6YD UK; 3 Nuffield Division of Clinical Laboratory Sciences, UK Department of Clinical Biochemistry Level 4, University of Oxford, John Radcliffe Hospital Headley Way, Headington, Oxford, OX3 9DU UK; 4 Department of Pharmacy and Forensic Science, Drug Control Centre, King’s College London, 150 Stamford Street, London, SE1 9NH UK

## Erratum

After publication of the original article [1], it came to the authors’ attention that there was an error affecting equation (7). A correction to this equation was submitted by the authors during proofing, but was not implemented correctly by the Production team.

The correct version of equation (7) is as follows:

7 $$ \begin{array}{l}\widehat{\beta^{*}}=\frac{{\displaystyle \sum_{i=1}^n\left({Y}_i^{*}-\overline{Y*}\right)\left({x}_i-\overline{x}\right)}}{{\displaystyle \sum_{i=1}^n{\left({x}_i-\overline{x}\right)}^2}}=\frac{{\displaystyle \sum_{i=1}^n\left({Y}_i-\widehat{\beta}{x}_i-\left(\overline{Y}-\widehat{\beta}\overline{x}\right)\right)}\left({x}_i-\overline{x}\right)}{{\displaystyle \sum_{i=1}^n{\left({x}_i-\overline{x}\right)}^2}}\\ {}\kern2.5em =\frac{{\displaystyle \sum_{i=1}^n\Big({Y}_i-\overline{Y}}\left)\left({x}_i-\overline{x}\right)-\widehat{\beta}{\displaystyle \sum_{i=1}^n\Big({x}_i-\overline{x}}\right){}^2}{{\displaystyle \sum_{i=1}^n{\left({x}_i-\overline{x}\right)}^2}}\\ {}\kern2.5em =\frac{{\displaystyle \sum_{i=1}^n\Big({Y}_i-}\overline{Y}\Big)\left({x}_i-\overline{x}\right)}{{\displaystyle \sum_{i=1}^n{\left({x}_i-\overline{x}\right)}^2}}-\widehat{\beta}\frac{{\displaystyle \sum_{i=1}^n\Big(}{x}_i-\overline{x}\Big){}^2}{{\displaystyle \sum_{i=1}^n{\left({x}_i-\overline{x}\right)}^2}}=0.\end{array} $$

Equation (7) has also been updated in the original article in order to rectify this publisher’s error.
